# Urgent Considerations on the Relationship Between the Advance of Covid-19 in Indigenous Territories in Brazil and the Impacts of Monoepistemic Public Policies

**DOI:** 10.3389/fsoc.2021.623656

**Published:** 2021-05-03

**Authors:** Alexandre Herbetta, Taís Pocuhto, Maria do Socorro Pimentel Da Silva, Cintia Guajajara

**Affiliations:** ^1^ Núcleo Takinahakỹ, Universidade Federal de Goiás (UFG), Goiânia, Brazil; ^2^ Indigenous School 19 de Abril, Goiatins, Brazil; ^3^ Faculdade de Letras (Liberal Arts Faculty), Núcleo Takinahakỹ, Universidade Federal de Goiás (UFG), Goiânia, Brazil; ^4^ Brazilian Indigenous Movement, Maranhão, Brazil

**Keywords:** pandemic, territory, public policies, epistemologies, COVID-19, state

## Abstract

This paper seeks to deal with the advance of Covid-19 in indigenous territories in Brazil, whether urban or rural. To do so, we have gone through a general analysis of the Brazilian government's indigenous policies, comparing bulletins and data from the Special Secretariat of Indigenous Health—Secretaria Especial de Saúde Indígena, an agency linked to the Ministry of Health, as well as data from the Articulation of Indigenous Peoples of Brazil, the main Brazilian indigenous political movement. Furthermore, we systematize strategies that have been developed and executed by some indigenous peoples in Brazil, undertaken by an exploratory analysis of manifestations of indigenous leaders on the internet, along with actions in the legal sphere, as well as, actions in the indigenous territory. Finally, the monoepistemic character of public policies on the issue is problematized.

Nature moves on. The virus does not kill birds, bears, any other beings, only humans. Those in panic are the human people and their artificial world, their way of working it is what is in crisis (2020, p. 44). Ailton Krenak. intellectual Brazilian leader from Krenak people.

Kaa ijasò mahãdu rèsèrèri ixỹ ube—ki, bèra ldu rỹirèri, utura, nawii kia tyytby ryirèrimy ihãre. Kiatahè tii mahãdu wna iny raxiwè mahỹre irèhèmy iny tarùmy rỹikèmy, bdè bdè rỹira tule irùmy rỹira kèmy. Ijasò hèka dèysa iny dèè riwymyhỹre, awimy iny ratxikèmy, iny rexibutunymy irùmy iny tabdèdỹỹnanadi rỹikèmy. Tii boho hèka aõni awi rare iny dèè. Bdèbdè roimyhỹre rexihukèki tahè ikymy òhutibèna rỹira rùsakè tahè iny boho ihèki rekexihurènykè.

Umya Karajá

## Introduction

This paper relies on the advance of Covid-19 in indigenous territories in Brazil,^1^ whether urban or rural from the beginning of the pandemic period to the end of 2020. This text aims at providing a better understanding of this situation, in order to lead to appropriate public policies and actions.

The writing is produced collaboratively by indigenous and non-indigenous intellectuals and thus, it stands for an indigenous struggle against Covid-19, based on political articulation and the search for alliances through collective action. It is important to emphasize that this text itself is also an indigenous report, since it is based on the authorship of indigenous and non-indigenous intellectuals.

Furthermore, the text also points out to collaborative methodologies developed in Latin America. In this way, the use of the pronoun “we” demonstrates the existence of a collectiveness composed by different subjectivities, which on this textual structure (and in behaviors) are placed in the same vibration and perspective, in despite of their differences. The main challenge of collaborative methodologies is precisely the creation of other subjectivities and spaces of interactions that turn individuals into collectiveness promoting life-changing for all those involved.

Our text is based especially on the experience of some particular indigenous peoples involved in the project of the Takinahaky Center of Higher Education for Indigenous Peoples, at the Federal University of Goiás, such as the Krahô, Apinajé, Tapirapé, Xerente, Guajajara, and Karaja People, just a sample among of the more than 300 different indigenous Peoples living in Brazil. Accounting for all of his diversity should be quite impossible for the purposes of this paper.

Furthermore, we take as our text basis, actions and perspectives of the indigenous movement in general, especially through APIB—Articulação dos Povos Indígenas do Brasil.

Regarding to the indigenous health, the National Policy of Health Care for Indigenous Peoples is part of the National Health Policy, and of the Organic Health Laws, along with the guidance stablished by the National Constitution, which recognize (or it should) the ethnical and cultural specificities, and the territorial rights of indigenous peoples (National Policy of Health Care for Indigenous Peoples, 2019, p. 1). The biggest issue has to do with the impacts of the monoepistemic features taken by public policies, since the national policies seems not to consider the differences emerged from and demanded by different Peoples.

According to Ouriques Ferreira (2013),

Since the 1970s, the World Health Organization (WHO) has recommended that national states integrate traditional medicines (TM) into their official health systems […] Only in 2002, however, when the WHO (2002) published the WHO Strategy Paper on Traditional Medicine, 2002–2005, it defined guidelines for establishing cooperative relationships between official health systems and practitioners of TM as a way to expand coverage and access to primary health care services for the population of developing countries (p. 205).

The indigenous health system is composed of Distrito Sanitário Especial Indígena (DSEI) coordinated by the Secretaria Especial de Saúde Indígena (SESAI) under the Ministério da Saúde (MS). The DSEIs coordinate the health local professionals, supporting the local heath teams responsible for low-complexity care at indigenous territories. For more complex occurrences, there are the regional or district hospitals. Highlight that a good part of the Brazilian indigenous communities is located at a great distance from those hospitals, hence another difficulty for the treatment of Covid-19.

In addition, traditional medicine is often not taken into account in institutional therapy and elders, for instance, do not trust the treatment of non-indigenous medicine. They complain, for instance, about the loneliness they experience while laid up in hospitals, meaning an intercultural silencing.

Focusing on indigenous societies, Langdon (2004) also states that,

In the case of Brazil, the indigenous health care model is characterized by a process under construction, which specificity is the result of the intersection of historical and political factors carried out since the 1980s with the implementation of the Unified Health System (SUS), the Brazilian Constitution of 1988, which recognizes the pluri-ethnic aspect of the country, and the growth of indigenous organizations. Since the 1st National Conference on Indigenous Health, the Ministry of Health has been striving to structure a unique indigenous health subsystem, nevertheless, integrated to SUS, considering the principles of social control and community participation in it, while respecting and articulating to the ethnic specificities of the groups covered. This model has undergone several changes, including two government decrees, dated from January 2004, which modify its structure. The indigenous health policy is in constant negotiation (…) (p. 30).

It is also important to consider that the impacts resulting from the advance of the disease at the moment are both broad and complex going beyond health and sanitary issues, also laying on territorial, educational and warranty of rights. Indigenous schools that seek to address the issues of the contemporary world, for example, are on hold, and many territories are being invaded by loggers, miners and evangelizers. As well as in Vera Cruz/Mexico, accordingly to Dietz and Cortés (2020, p. 1) intercultural schools are been closed affecting the lives of indigenous children and teenagers.

Furthermore, we must acknowledge that the fight against the invisible enemy is taking place, nowadays, in indigenous populations across Latin America. Mexico exemplifies a similar situation.

Cherán, in the same way that the rest of the indigenous communities of the country is experiencing the pandemic. Public health issues, the recession of the local economy and psychological effects are some of the problems facing the community. In the community imaginary the questions arise: how long will it last? And how are we going to face it if we do not have the medical infrastructure to deal with problems of this nature? (Jiménez et al., 2020, p. 1).

The challenge facing the indigenous Brazilian Peoples is enormous, not all people respect the local indigenous laws nor the national ones. Covid-19’s impact on indigenous territories and spaces is threatening and will certainly remain within the collective memory of these peoples. Nowadays, we are neither able to sleep well, nor visit the relatives and beloved ones. Our ways of living are severely impacted. Besides that, the indigenous languages are being suffocated by the majority languages, deforestation, the death of rivers and lakes, the extinction of animals, the decreasing in fish, fruits, insects, etc. The words of those languages, as well as their meanings and senses stand on nature, understood here outside the western dichotomy nature and culture. All of this also contributes to the weakening of mythologies. Besides Covid-19, indigenous peoples are facing many other pandemics.

All of this let us question, for instance, what is the real situation of the native peoples of Brazil regarding this pandemic? What about the defense of our territories, our rights, and upon the intercultural education? What about the health of our population? Why are we sidelined, once again, in relation to the effectiveness (and appliance) of official policies? Why do we remain vulnerable? How will we be after this pandemic? How can we have intercultural educational systems and policies based only on Western knowledge? Not without articulating knowledge needed for indigenous struggle; policies to value indigenous languages; epistemic bilingualism; indigenous epistemologies; considering the ways in which indigenous peoples organize their knowledge; understanding that the grammar of indigenous languages is culture, art and nature.

## Discussion

### Underreported Data, Ineffective Policies

**FIGURE 1:**
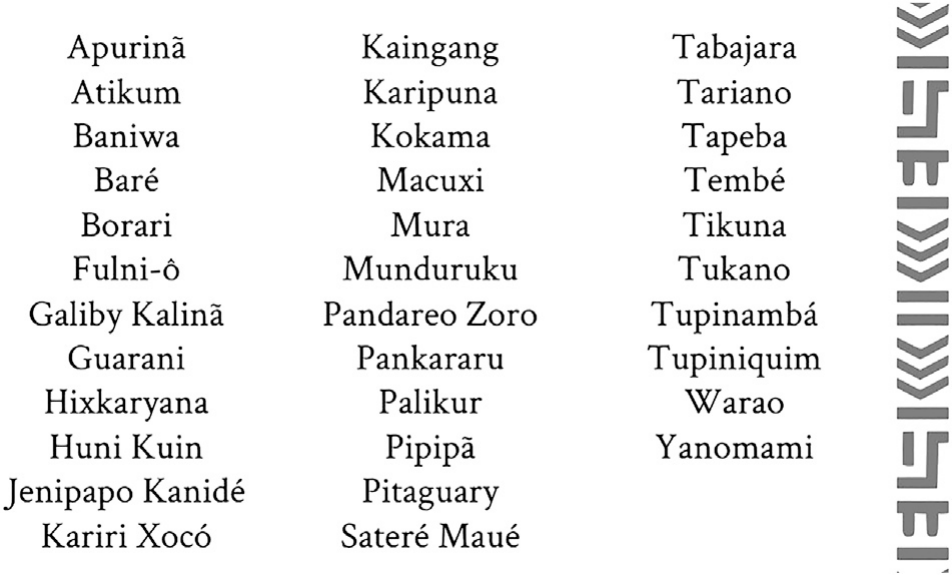
Instituto Socioambiental, 2020.

**FIGURE 2:**
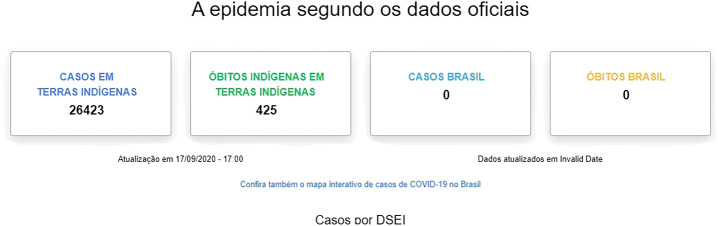
https://covid19.socioambiental.org/.

**FIGURE 3:**
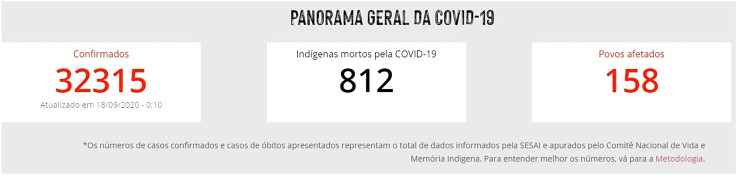
http://emergenciaindigena.apib.info/dados_covid19/.

First of all, attention should be drawn to the disparity between the official data collected on the topic presented by SESAI/MS and the data presented by other non-governmental bodies and institutions. Figure 1, below, indicates some of the many indigenous peoples impacted by Covid-19. The indigenous movements, for instance, into dialogue with the Articulação dos Povos Indígenas do Brasil (APIB) claim that the cases of contamination and deaths are much higher than the official data, which is critical, once it makes up the real situation. According to the National Committee for the Life and Memory of Indigenous Peoples, an entity related to APIB, there is a crucial issue of underreporting. The disparity is promptly depicted below (Figures 2, 3) is daily updated by the sources mentioned.

The difference in methodology explains such distinction. While this committee includes both indigenous people who live in traditional territories and those who live in an urban context, as provided by Convention no. 169 of the ILO (based on self-declaration and community ties), SESAI/MS does not register indigenous people living in an urban context, which is strongly rejected by APIB. This “requires the urgent revocation of ordinance 070/2004 to ensure that SESAI be of use of all indigenous people” (Indigenous Emergency, 2020).

The fact that indigenous people in an urban context are not considered by SESAI became clear on April 3, 2020. According to the news at the ISA Socio-Environmental Institute, “SESAI denies health care to indigenous people living in cities—Indigenous Health Secretary Robson Silva says that only indigenous villagers will be assisted at SESAI. This excludes 324,800 who live in cities. For indigenous organizations and the MPF all indigenous people should be assisted” (Instituto Socioambiental, 2020).

In order to reach such numbers, the committee’s data collection is decentralized through access to various grassroots indigenous organizations, including SESAI, Municipal and State Health Secretariats and the Federal Public Ministry (MPF). Updates happen on daily basis and data release only take place based on the consolidation from the previous day information. For the same institution, “due to the lack of transparency and the lack of details on SESAI’s information, it is not possible to check duplicate cases between the two databases. The number presented represents the sum of the data reported by SESAI and verified by the Committee” (Indigenous Emergency, 2020).

This underreporting covers up the situation, making invisible the dramatic nature of the issue, the urgency of action and the depth of the moment. It prevents an adequate analysis, making the elaboration of public policies insufficient and out of time. Many indigenous people also remain on the margins of more adequate treatment. It should be noted that the underreporting situation is observed in relation to quilombola peoples (afro-descendant people that lives collectively) as well. For Arruti,

Underreporting has become a central matter in addressing the Covid-19 pandemic in Brazil. However, if the situation of underreporting is serious in the big metropolises and aggravated in their outskirts, what to think of the rural communities? And if we add to this underreporting plan the effect of vulnerability and invisibility of the black population, as already demonstrated in the government resistance in introducing race and color information in the Ministério da Saúde (MS) updates? In fact, even after being introduced in the collection tools, they remain inaccessible in the government portal that publishes data about the advance of the disease. (Arruti, 2020, p. 1)

### Insufficiency of State Action

Hence, facing the unawareness of the real situation, as explained above, there is a consensus in the indigenous movements and in sectors close to them that the federal government has failed to take adequate action in relation to the advance of Covid-19 in indigenous territories. If we think about an indigenous policy carried out by the Brazilian government on the subject, it is noticeable that basic support measures were not taken in time to control the advance of the disease, such as mass testing and the cession of equipment and professionals, as well as planning the whole action. It should be noted that the temporality of the policies is fundamental to save lives and avoid the circulation of the disease.

The first case confirmed at an indigenous context took place in april. On april 1st, according to the website Agência Brasil, the first confirmed case of contamination of an indigenous Kokama woman was reported who, accordingly to the source, may have happened precisely when in contact with a doctor in the health system, who should have been away.

The Health Surveillance Foundation of Amazonas confirmed today (April 1st) the first case of the new coronavirus among Brazilian Natives Indigenous. According to the foundation, linked to the state health secretariat (Susam), this is a 20-year-old girl of the Kokama ethnic group who works as an indigenous health agent in the region of the city of Santo Antônio do Içá (AM). The municipality, which is located in the micro-region called Alto Solimões, is part of the Alto Solimões Special Indigenous Health District. According to the foundation, the patient may have had contact with the doctor who works in the same district and who also tested positive for Covid-19 (Rodrigues, 2020).

In the same way, according to a report of the Instituto Socioambiental on June 22, 2020, the National Indian Foundation (FUNAI) did not execute the budget for Covid-19 with the necessary urgency. According to the text “FUNAI have received more than 11 million emergency resources for the protection of indigenous peoples, but spent less than half of this amount (39%)” (Instituto Socioambiental ISA, 2020). Hereupon, for Verdun,

A significant part of Covid-19’s impacts on indigenous territories stem from this neglect of indigenous health. But let's face it, just increasing the budget is not enough. It is necessary to create mechanisms that control possible deviations of purpose, overcharged expenses, and the privilege of compatriots in the use of resources (Verdum, 2020, p. 37)

**FIGURE 4:**
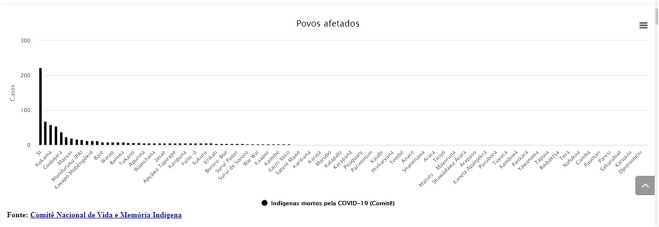
Comitê Nacional de Vida e Memória Indígena http://emergenciaindigena.apib.info/dados_covid19/.

Over time more than a hundred indigenous peoples have been affected by the pandemic at different levels, as Figure 4 bellow points out. The graph below shows the evolution of the situation.

October was the month that this text was produced and policies needed to restrain the disease and to support it are only timidly outlined, without at least a concrete plan established, which evidently worsens the situation.

Many indigenous leaders call attention to this lack of planning and organization to get aid in the city, for example, which ended up leading to contagion. For a Tapirapé teacher, there are cases of contagion because of contact within the city, because there was no precaution from government agencies about the access to benefits and other demands that must be carried out in the city, leaving aside the necessary particularity of the indigenous health policies, as it was mentioned.

In this dramatic scenario, crimes against the indigenous territories such as deforestation, burning and invasions have increased.

Nevertheless, Cerrado has been the biome that recorded the highest number of fires on indigenous lands in 2019, as occurs historically. The 9,543 outbreaks of fire in 2019 were about nearly twice more than the ones recorded in 2018 and, as in the Amazon, were 17% above the average recorded in 2009. The biome that had the largest increase in fire outbreaks on indigenous lands in 2019 was the Pantanal. Along 499 records, indigenous lands in this biome burned almost seven times more than the previous year. The number is also 3.7 times higher than the average recorded between 2009 and 2018. Mato Grosso do Sul, the state that covers most of the Pantanal, registered, in 2019, an increase of 452% in the outbreaks of fire on indigenous lands. The Indigenous Land (TI) most affected in the state - and the second most affected by fires in the country - was Kadiwéu, which is located in the borderline of the Pantanal and Cerrado and registered 1,268 outbreaks of fire. For some years now, the Kadiwéu have been denouncing the private appropriation of the territory by landowners and demanding from the State for the keeping off the area, which is regularized (Rangel, 2020, p. 24).

The Yanomamis report the presence of about 20,000 miners within their territory, increasing deforestation, pollution of their rivers and the danger of contagion. According to the Violence Against Indigenous Peoples Report organized by the Conselho Indigenista Missionário (CIMI).

The monitoring of satellite images by Instituto Nacional de Pesquisas Espaciais (INPE) revealed an increase in deforestation in the Amazon in 2019 caused by illegal gold mining. Together with the Kayapó and Munduruku peoples, the Yanomami are deeply impacted by this criminal activity. Indigenous peoples estimate there are about 20,000 miners within the Yanomami IT […]. Information by BBC Brazil, July/2019 (Rangel, 2020, p. 84).

It should also be mentioned that crimes against the lives of indigenous peoples have also intensified. As can be seen, the increase in attacks on indigenous leaders has grown substantially over the past year.

Data, from the state itself, show the murder, in 2019, of 113 indigenous people in Brazil. Many of them were leaders who put their ideas, proposals, and even their bodies in defense of their rights and protection of sacred lands, demanding demarcation and territorial regularization, opposing a government of predators and devastators of lives, human lives, lives of forests, lives of animals, lives of all beings (Rangel, 2020, p. 1).

In this context, according to Baxy Apinajé, the support for the actions of her people to control the disease came from the municipality of Tocantinópolis/TO by offering an isolation house in the city (Baxy, 2020a; Baxy, 2020b). The municipality also collaborated with inputs for the sanitary barriers effective enough to ensure the isolation of the villages. Furthermore, the Karajá people has received aid from the municipalities, solidarity from non-indigenous, and through the SOS/KARAJÀ organization, and they obtained inputs for the sanitary barriers and rapid tests, donated by the Universidade Federal de Goiás (UFG), and resources to buy medicines donated by ABRALIN.

### Action, Indigenous Protagonism and Collectivity

In this context of underreporting and lack of governmental action, leadership and indigenous community meetings, often carried out in the central courtyards, are fundamental spaces for the fight against the pandemics. The performance of chiefs, shamans and leaders is central to indigenous resistance. This struggle is added to the efforts of indigenous and non-indigenous health professionals who give themselves intensely to contain the advance of the pandemic.

These meetings and articulations are fundamental for the execution of actions. The Krahô people, for example, hold several meetings with leaders, chiefs and relatives in the Ká (central courtyard) so that all villagers act with cohesion to strengthen the fight against this invisible virus. Regarding the situation of quilombola communities.

The complete lack of knowledge about the impact of covid-19 on quilombola territories only starts to disappear at the initiative of the quilombola political organizations themselves in partnership with universities and civil society organizations. As in the metropolitan suburbs, it is a question of resorting to the logic of “we for ourselves”. The main initiative in this sense came from the partnership between CONAQ (Coordenação Nacional de Articulação de Quilombos) and ISA (Instituto Socioambiental) in the creation of the Observatory platform of Covid-19 in Quilombos. Such initiative is a denunciation of the invisibility of the pandemic in these communities, while taking the lead in its monitoring. The platform performs an updated count of the number of cases monitored, confirmed cases and quilombola deaths, as well as the projection of this information on an interactive map, in which it is possible to locate the communities with cases and the nearby public hospitals with ICU. It also offers an updated list of news on the subject (Arruti, 2020, p. 1).

In the indigenous territories, one of the most successful actions was the health barriers. Among the Krahô, for example, a sanitary barrier was created at the main entrance to the city of Itacajá in order to prevent the exit of the *mehis* (indigenous people) to the city and, at the same time, to prevent the entrance of strangers at the reservation area. The indigenous warriors were chosen to be at the gate to watch over the day and night. However, they have suffered death threats because they have been preventing the entry of indigenous people into the city, which shows the complexity of the situation.

Among the Apinajé people,

The Indigenous Sanitary Barriers are at the limits of the territory. One is at the border with Tocantinópolis, within our territory, on the Transamazon highway. This barrier holds 23 villages. There is another sanitary barrier that is located on the TO-126 highway, which connects the cities of Tocantinópolis and Maurilândia. And another barrier at the entrance of the Prata village which has entry through the road and the Cocal Grande village, guarding another 26 villages. The guardians thus closed the two ends of access to the territory, on the side of the Mariazinha village and the side of the São José village. We made the protection circle of the villages (Baxy, 2020, p. 5).

The relationship with the city is therefore an important element in the context and, in this case, is quite intense, the city being often an abusive space, which discriminates, even though benefits from the indigenous presence. This is true because in some communities many things are needed from the city. Baxy Apinajé, for example, states that “surrounded by cities, soy, plantation, eucalyptus, the Tocantins River with a lot of dams, there is no more fish. We go to the city for supplies, for sustenance” (Baxy, 2020).

In order to protect themselves, several indigenous people chose to be in isolation in their fields to keep distance from others and they not need the relationship with the city, however many don't stay for long and end up returning to the village.

APIB and other indigenous movements also act strongly at the institutional level, accessing spaces in the Brazilian state. In this direction the articulation of the indigenous movement, through indigenous lawyers and in dialogue with political parties, strongly demands effective actions from the federal government. ADPF 709/2020 should be highlighted,

This is an allegation of non-compliance with a fundamental precept proposed by the Articulation of Indigenous Peoples of Brazil—APIB, by the Brazilian Socialist Party—PSB, by the Socialism and Freedom Party—PSOL, by the Communist Party of Brazil—PC do B, by the Sustainability Network—Rede, by the Labor Party—PT and by the Democratic Labor Party—PDT. The action has as object a set of commissive and omissive acts of the Public Power, related to the fight against the pandemic by COVID-19, which would imply high risk of contagion and extermination of several indigenous peoples, in violation of the dignity of the human person (CF, art. 1, inc. III), the rights to life (CF, art. 5, caput) and health (CF, arts. 6 and 196), as well as the right of such peoples to live in their territory, in accordance with with their cultures and traditions (CF, art. 231) (Fundamental Requirement Arguition 709 Federal District).

In this regard, APIB questions the country’s deficiency compliance with constitutional and fundamental precepts in combating the pandemic of the new coronavirus among Brazilian indigenous peoples. This statement disrespects the 1988 Constitution and international agreements, such as Convention 169 of the International Labor Organization, to which the country is a signatory. Accordingly, on July 8, the current Minister Luis Roberto Barroso of STF (Superior Tribunal Federal) agreed with the indigenous demonstration and stipulated that the State must take concrete and effective action against the advance of the Covid-19 pandemic among the indigenous population. The judge based on part of the indigenous rights, especially articles 231 and 232 of the National Constitution. The first emphasizes the need to pay attention to the indigenous cultural particularities and the other guarantees the originating peoples the possibility of legal representation in relation to the Brazilian state.

Moreover, a Joint Parliamentary Front in Defense of Indigenous Peoples was organized at the institutional level, led by Federal Congresswoman Joênia Wapichana, who is attentive to government actions and seeks to propose appropriate legal measures to address the issue. Noticeably this Front, in partnership with APIB and other indigenous movements, in the absence of a state plan, assembled the document “Indigenous Emergency” (http://emergenciaindigena.apib.info/), an action plan to deal with the dramatic situation, based on guidelines, actions and communication strategies.

It is time to reflect on the way of life that we have cultivated until the present day, because the various environmental crises and catastrophes are the result of actions with strong impacts on the environment that lead us to the advance of global warming, loss of vegetation, biodiversity and other profound changes in nature. They are the harbinger that we are living today, alerts from mother earth about our way of existing, which needs to be rethought. It is clear to us that we need to exercise even more solidarity. For Brazil and the world, this viral war may be new, but for us indigenous peoples it is not. We already know this reality because we were victims of the diseases used as a strategy in the process of invasion and colonization of Brazil, as well as other aggressions, such as those practiced during the military dictatorship and in the present times to exterminate our peoples, our identity and our way of life and usurp our territories, besides the natural goods that we have preserved for millennia. In Brazil we are going through difficult days, of much sadness and political uncertainties, there are already more than 43 thousand lives lost and 800 thousand infected by Covid-19, with a lethality rate of 6.6% in the general population. And we are all finding that Covid-19 affects the indigenous peoples even more lethally. By June 16, 287 indigenous people had died and 5,484 were infected, with 103 people impacted in more than 17 states of the federation. The power of the virus’s lethality among indigenous peoples is above that of non-indigenous peoples, which demonstrates its strong capacity for destruction (National Plan - Indigenous Emergency, 2020, p. 7).

The communication and information strategy seems essential in this period. APIB continues to communicate the situation equally and intensely publicly, whether in its pages http://emergenciaindigena.apib.info/dados_covid19/, virtual events such as “webnaries” or lives, campaigns such as “Indigenous Lives matter”, or producing information and media in an efficiently as in multilingual bulletins (Portuguese, English, Spanish, French and Italian) or at http://emergenciaindigena.apib.info/files/2020/06/01-Card-Quarentena-Indigena-PT.pdf.

Likewise, there is a range of fundraising campaigns for the maintenance of sanitary barriers and the purchase of equipment as well as the donation of inputs needed for survival. Clearly the pandemic and social isolation there is an impact on income generation of indigenous peoples and the relationship with the city, where many families today complement their farms.

The Save Krahô campaign, for example, aims to support the indigenous Krahô people in facing the Covid 19 pandemic. Its actions consist of: ensuring the maintenance of guardhouses at the entrances to the Indigenous Territory, preventing non-indigenous people from accessing the villages and ensuring that everything that comes from the city is properly sanitized; providing protection and hygiene equipment for the indigenous people who need to go to the city and bring information to all the villages. There are also projects that seek to strengthen private and community farms.

Therefore, despite the isolation of the communities, a key strategy in the fight against the advance of the disease, it is perceived that a collective articulation is being sought in a virtual way, strengthening an approach of collective through the participation of indigenous, non-indigenous people, leaders, artists, intellectuals, Brazilians and people from outside the country. There is also an interesting articulation between indigenous peoples.

### Amerindian Ancestral Memories and Cosmopolitics in Action

Another substantial aspect to consider is that the devastating impacts of a pandemic are part of and also access the collective memory of many indigenous peoples, who have experienced similar situations in past decades, producing psychic struggle.

For Jiménez et al. (2020),

In the collective memory of the community are the stories of the Spanish fever that spread in the second decade of the 20th century. Grandparents say that dozens of Cheranenses died from the disease. For fear of contagion, some fled to the hills to take refuge, others stayed at home without going out so as not to get infected. One of the main characteristics was high fever, colloquially the disease was known as “fever” (2020, p. 1).

Shiela Baxy Apinajé, in an article, recalls that the first situation lived among the Panhi Apinajé people at this time was that of psychic suffering.

We began to experience a psychological outbreak of the disease, because the Apinajé, in their memories, had already passed through another epidemic. We remember that in the outbreak of smallpox (Spanish flu) in 1808 many indigenous people died, almost leading our people to extinction. It was a very violent moment for our people (2020, p. 3).

Teacher Koria Tapirapé also mentions that death from infectious flu and malaria diseases in the 1930s and 40s is now a reality narrated by the elders, who claim that the Apywa population was only 52 individuals. This process is also painful and causes fear.

The virus intensely also affects the coexistence of people who are prevented from practicing their rules of coexistence, which almost always tend to the collective and reciprocity. For Sheila Baxy Apinajé the coronavirus attacks the indigenous culture (2020). For Professor Silvino Xerente the indigenous culture is that of agglomeration (Professor Silvino Xerente, 2020).

If on the one hand it affects the Amerindian cultures, on the other hand the fight against the Covid-19 accesses the various Amerindian cosmopolitics. Still on Sheila Baxy,

For older people this new disease is like mē à, known by the name of kupyt kak, which is the disease of the guariba monkey, which brings very strong flu, very high fever and can kill very fast. The elder José Ribeiro (Zé da Doca) says that the disease has no rattle to warn us and that it is confusing, attacking the psychological side, because the non-indigenous people are dying and we may disappear from the earth (2020, p.3).

Similarly,

Cherán/México, as an indigenous community, strengthens its community cohesion through its rituals, festivals, assemblies, uses and customs, which is why the isolation and social distancing measures promoted by official health organizations seem contrary to community life (Jiménez et al., 2020, p. 1).

Dietz and Cortés note the same

In several regions of Veracruz, as also happens in other Mexican states (Jiménez et al., 2020), indigenous communities reacted to the pandemic in culture specific ways, not by passively confining themselves to individualized, fragmented domestic units, but by closing off all access to their community as a whole, so as to remain not at home, but in their community. These bottom-up local strategies, which the communities even managed to impose on their own local neighbors who were trying to return home after losing their jobs in urban or tourist areas, in order to (2020, p. 4).

The resistance to the advance of the pandemic is then in the knowledge of the elders, in the therapeutics of the bush and in the indigenous spirituality. Koria Tapirapé states that “from our joint struggle we have the prohibition of some food, usually the strongest meats, considered as bearers of evil spirit. The consumption of these meats could make the situation worse, as cosmology explains” (Valdvane Tapirapé, 2020). To him, yet,

a notable and interesting element is the access to the different spiritualities, we did this to add the strength of our protection, with the apyawa cosmology, with the owner of the river, the fish, the hill of animals. It is a way to cheer our spirits to protect us from the pandemic. We count on the strength of our aremomoya, a big person, who takes care of the organization of nature, we have supernatural beings to overcome the pandemic, according to cosmology so that the disease comes with less aggression” (Valdvane Tapirapé, 2020).

Umya Karajá says that for his community, the songs of the Ijasò practiced in these difficult times contribute to warding off the virus. He says, “We are together praying for Arowans to continue to exist. Arowans bring joy, peace, togetherness and a lot of cultural motivation. They are important to our Iny community. If our nature, which is a great defender of various diseases today, ends, we will all end up together” (2020).

Many people always emphasize the use of traditional medicine, even if it is not properly respected by the Brazilian health system, as already aforementioned. For the Krahô peolpe, “our doctor was the shaman himself” (Pohcuto, 2020). For Koria Tapiarapé “we can never think of commercializing traditional medicines. It does not compact with Mayra (non-indigenous) medicines. The owner himself takes away the power of medicine. It would be without effect. It would not cure anything. In this sense we had medicine for everyone. The shamans have made medicine available to everyone. It was a collective struggle (Valdvane Tapirapé, 2020).

A similar attitude was adopted by the Karajá people. Kuriawa, a great teacher and promoter of the culture of his people, told us that his community was treated with by means of medicinal plants. For him, medicinal plants have the power to ward off the corona virus. He celebrates the healing by thanking nature. In general, we have realized the power of healing through the spirituality of medicinal plants among indigenous people. If it were not for the power of medicinal plants of indigenous wisdom, the disaster would be much greater. This, however, does not exclude the possibility of extinction of small population indigenous peoples.

The extinction of a people represents the extinction of its language and the irrecoverable loss of its unique cultural, historical and ecological knowledge. It means the loss of some humanity present on the planet. It is unrecoverable.

The Tenetehar people, also known as Guajajara, from the state of Maranhão, as well as other indigenous peoples, were not prepared to face this disease. When news broke on television that a terrible virus was spreading rapidly around the world and that it would soon reach Brazil and indigenous territories. They clung to their ancestral prayers and went to seek help in nature and in ancestral memory. Cíntia Guajajara’s mother recalled the hot herbal teas she had learned from her grandparents, the seeds and water from the bark of quina, which she had learned from her mother.

They bathed the children with these herbs. Coumaru and saffron helped a lot in fighting the virus. They fight the virus through medicinal plants. They went back to eating food from their region: native fruits, food that comes from the fields, such as corn, beans and potatoes. They also fish from their river again.

Daily Guajajara people made tea for the community; everyone drank. They reinforce the power of plants, seeds, tree houses, food from the fields. That’s how they controlled the virus. They made ointments (creams) from the leaves of the trees. The leaf baths of the “black mine” also helped a lot. Their ancestral science saved them from this terrible virus. They remain vigilant and taking care of themselves. According to Cintia Guajajara, she

cried a lot at each loss of a relative, I suffered a lot, without being able to help the more distant relatives, because we had to stay away, when I knew that the relative had the virus it was too late. Many avoided going to the doctor, looking for alternatives in the sacred medicinal plants Guajajara (2020).

Koria Valdvane Tapirapé also reinforces the intention to the elder’s importance: “It is they who carry our epistemologies, our logs, our readings about nature and the world. We have lost an elder, we have lost a set of knowledge and wisdom. This is the most critical point” (2020). Manaije Karajá embodies his pain by speaking of the deaths of many wise men and women, guardians of millenary knowledge and great masters responsible for transmitting specialized knowledge to younger generations. In their community, many women experts in moving knowledge of their people through art died. These women were mothers of Karajá teachers.

Throughout Brazil, countless indigenous teachers also died, victims of the Covid-19, many of them were also leaders, and fighters of their peoples for the defense of indigenous knowledge and languages.

## Results

### Monoepistemic Necropolitics

**FIGURE 5:**
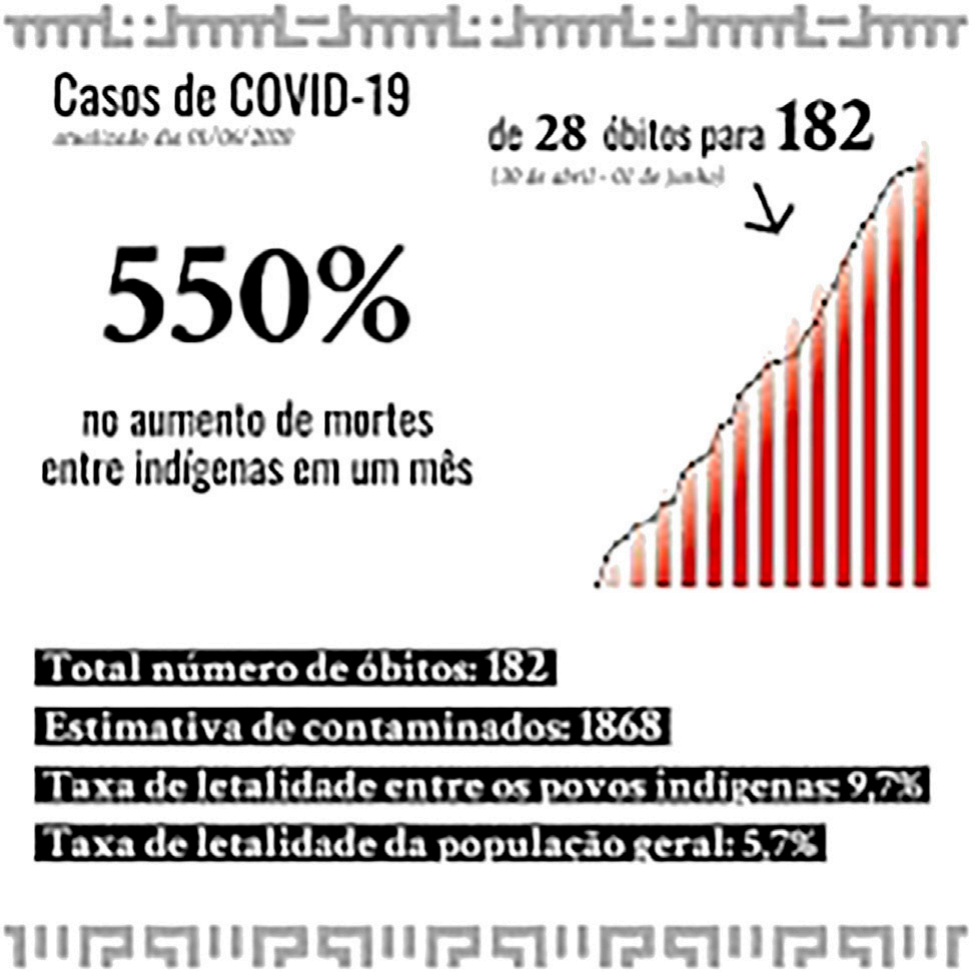
https://www.facebook.com/apiboficial/photos/pcb.2597742657162554/2597742457162574/.

As can be seen, Covid-19 continues to advance over native territories and affecting indigenous villages or indigenous peoples in an urban context. This is happening under strong indigenous resistance, and is based on their epistemologies and cosmopolitics. The lethality rate among indigenous peoples remains much higher than for other peoples (Figure 5 below) in the country and abroad, which concretely shows that the situation continues to eliminate indigenous bodies and knowledge.

In accordance with Jimenez; Fernando; Juarez, in Mexico,

The pandemic has demonstrated the inability of the State to resolve the public health issue of the P’urhépechas people. In this sense, the community of Cherán faces serious problems of chronic degenerative diseases: diabetes, hypertension and obesity in adult population that ranges between 22 and 70 years of age. Diseases are the product of changes in the food and health habits in the daily life of indigenous people. On the other hand, COVID-19 shows the vulnerability of the community to the pandemic that has wreaked havoc on this sector of the population in large cities. In addition to this, there is concern that the medical infrastructure to deal with it is weak. If there is an outbreak of COVID-19, the probability of an infectious condition with devastating consequences for community members is high (Jiménez et al., 2020, p. 1).

Within the same context, Arruti

add to these variables the historical-structural racism that kept most black rural communities out of the field of investments and expansion of state and municipal public health policies. Thus, we will understand why quilombola communities are in an extreme situation with respect to the issue of underreporting and, most likely, in terms of vulnerability to the advance of the pandemic. The precarious situation of these communities in relation to land and educational policies has been widely addressed by specialized literature. Although the pandemic has alerted us to the need and urgency to shed light on this precariousness, concerning access to health policies. In this context, the idea of precariousness is confused with that of necropolitics, that colonial and post-slavery variation of biopolitics: a way of governing life that normalizes the death of some in favor of the security of others (Arruti, 2020, p. 1).

As a result of state policies, indigenous and black bodies continue to be more affected than others. For a Xerente teacher,^2^

our authorities who are in front of the Ministério da Saúde (MS) lacked assistance. We thought about how the Akwe people will be served. I even received the mask only once, I didn't receive alcohol gel, I didn't receive anything. I wonder if this is prevention? We need to be better served; we need our authorities differentiated service for our people.

According to another Tapirapé scholar

the government is genocidal, ecocidal, gerocidal, it is the government that practices necropolitics. Our right is being violated, in the constitution and in international law. The government does not take the slightest consideration towards the indigenous people. There is only hate plan. The government that preaches hatred, prejudice, discrimination, intolerance over us. It is the government of authoritarianism. More than 500 years have shown our resistance, our struggle, transmitted by the wind, according to shamans.

The assumptions of necropolitics in agreement with Achile Mbembe is the state’s ability to define in its actions who lives and who dies. For the author, the assumption “is based on the concept of biopower and exploits its relationship with the notions of sovereignty (imperium) and the state of exception” (2016, p. 124). For the same author, it is assumed “that the maximum expression of sovereignty lies, to a great extent, in power and the ability to dictate who can live and who should die” (p. 123).

Undoubtedly one of the strongest and most evident of necropolitics is its monoepistemic foundation. At the same time, it eliminates bodies, dismantles knowledge, unties relationships, and attacks other ways of being and knowing. For Mbembe “disregarding this multiplicity, late-modern political criticism has unfortunately privileged normative theories of democracy and made the concept of reason one of the most important elements of both the project of modernity and the territory of sovereignty” (Mbembe, 2016, p.124).

Hence, the public policies in focus have been strengthened their monocultural aspect, intensifying centralized decision mechanisms and reproducing an authoritarian and violent power and decision structure. In other places the same thing happens. For Dietz and Cortés, in Mexico, “Unfortunately in Veracruz but also in the rest of the country, it seems that the pandemic has empowered centralized instances of educational, health and political decisions in general” (2020, p. 7)

This has even cause distrust among indigenous peoples regarding the treatment they will undergo in hospitals. Many do not accept being hospitalized, they prefer to stay in their communities, even at the risk of dying.

Similarly, to Damsokekwa, on education, everybody should understand the importance of having indigenous managers in occupying decision spaces (Damsokekwa, 2020, p. 5).

It is undoubtedly necessary to improve communication in the understanding of cultural diversity and multiplicity, which also means to perceive and reverse the systematic silencing of discourses that aim to deconstruct hegemonic thoughts and practices that exclude knowledge, languages and worldviews. The epistemic articulation allows building new communicative paradigms, even in health, education or any other area, as they require intercultural dialogue in priority tasks such as social emancipation, confronting inequalities without annihilating identity and differences.

As discussed throughout the text the main actions for resistance to Covid-19 and the treatment comes from indigenous peoples. More than ever institutions must be intercultural, with a critical character, making communication between epistemologies possible and reinforcing action in different cultures and contexts. For Damsokekewa, it is necessary to have indigenous agents in the decision processes (2020).

The Covid-19 will leave many narratives of pain and suffering as convey many narratives of revival of indigenous epistemologies. It will also leave the urgent need for collaborative methodologies in public administrations. Understanding indigenous strategies in the fight against the disease, through social organization, medicinal herbs, songs and prayers of indigenous (non-Christian) tradition to thank nature for the remedies has a meaning of greatness and make us reflect on the violent, mechanistic, rationalist and positivist bases of public policies. This same process has culminated in such destruction of ecosystems, widespread social collapse without finding harmonic points for the global human community.

This discussion demonstrates how policies need, in reality, to change their old paradigms, centered on the fragmentation of knowledge, on the rationalization of the world and on cultural and human distance.

The pandemics is indicating the need for more complex models of analysis and that the impact of an infectious disease in this portion of the Brazilian population is also associated with other particularities and contingencies as sociocultural, political, historical, food, nutritional, epidemiological, emotional, economic, territorial and environmental. The pandemic also spells out the weaknesses of the structured care system to protect and promote the individual and collective health for indigenous peoples, and how vulnerable and dependent it is on the (dis) commitment of public managers on duty with their rights, including healthy (Verdum, 2020, p. 35).

We suggest that public policies and State institutions should be anchored in co-theoretical and intercultural foundations, incorporating different languages and forms of expression in the dialogical process of managing demands. This saves lives. It is essential to develop collaborative methodologies in rescuing the silenced cultural links and erased epistemic spaces, The art, narratives and histories of oral literature contribute to the construction of new methodological and pedagogical approaches to be considered in the investigation and production of new epistemic bases.

All indigenous lives matter! All lives matter!

As a country, we need to recognize the epistemicide, as Correa Xakriabá (2018) argues, which occurs from the monopoly of monoepistemic policies which impacts the circulation and existence of other forms of knowledge that do not fit in the established canons of eurocentric knowledge. We must all put an end to the monoepistemic necropolitics that allows the death of many knowledges and bodies.

These points of reflection should be considered when facing pandemics and prejudices experienced by indigenous people in different dimensions of living with non-indigenous societies. We must have respect for the wise men and women of the communities and respect for the sacred medicinal plants. Never deforest, never burn! We must learn our and different knowledges even more. The ancestral knowledge can heal!

## Footnotes

1 According to the 2010 Demographic Census, Brazil has 896,917 indigenous people, with 517,383 people in rural areas and 379,534 in urban areas. There are more than 300 distinct indigenous peoples who speak 174 different indigenous languages.

2 The two indigenous leaders below preferred not to identify themselves for security reasons

## Abbreviations

APIB, Articulation of the Indigenous Peoples of Brazil (Articulação dos Povos Indígenas do Brasil); CIMI, Indigenous Missionary Council (Conselho Indigenista Missionário); CONAQ, National Coordination of Rural Black Quilombola Communities (Coordenação Nacional de Articulação de Quilombos); DSEI, Indigenous Health Districts (Distrito Sanitário Especial Indígena); MS, Ministry of Health (Ministério da Saúde); SESAI, Special Secretariat of Indigenous Health (Secretaria Especial de Saúde Indígena); STF, Federal Supreme Court (Superior Tribunal Federal).
